# Convulsions during cataract surgery under peribulbar anesthesia: a case report

**DOI:** 10.1186/1752-1947-8-218

**Published:** 2014-06-23

**Authors:** Mustapha Bensghir, Najlae Badou, Abdelhafid Houba, Hicham Balkhi, Charki Haimeur, Hicham Azendour

**Affiliations:** 1 Department of Anesthesiology Military Hospital Mohammed V Rabat, University of Mohammed V Souissi, Rabat, Morocco

**Keywords:** Brainstem anesthesia, Cataract surgery, Convulsions, Peribulbar anesthesia

## Abstract

**Introduction:**

Locoregional anesthesia techniques are increasingly used for cataract surgery. From these techniques, peribulbar anesthesia has been very successful over the retrobulbar anesthesia seen its effectiveness and safety. However, peribulbar anesthesia is not without risk.

**Case presentation:**

A 70-year-old African man was scheduled for cataract surgery and lens implant for his right eye. His medical history included hypertension, diabetes mellitus and gall bladder surgery. There were no personal or family antecedents of allergy, epilepsy or taking food or toxic drug. No abnormalities were detected in his preoperative evaluation. In the operating room, standard monitoring was installed and a peripheral venous catheter 18g was inserted. Peribulbar anesthesia was realized with two injections in primary gaze position. The anesthetic mixture contained lidocaine 2% and bupivacaine 0.5%. The needle used was 25GA, 19mm, ¾ inch. The first injection was performed in his lower temporal peribulbar space with 5mL of mixture; the second injection was performed with 3mL of mixture in his upper nasal peribulbar space. These injections were performed after a negative aspiration test and followed by manual compression of his globe for 5 minutes. Five minutes after peribulbar anesthesia, his blood pressure increased to 209/115mmHg requiring three bolus of nicardipine (3.0mg) to reduce his blood pressure to 134/56mmHg. One minute after, he had generalized tonic–clonic seizures. Tracheal intubation was performed. His capillary blood glucose was 170mg/dL, axillary temperature was 36.5°C, and his serum electrolytes were normal. He recovered spontaneous ventilation 1.5 hours later. A neurological examination noted no deficit. Extubation was performed 15 minutes later without incident. A brain computed tomography and electroencephalogram were unremarkable. He was discharged on the second day and operated on 1 month later under general anesthesia.

**Conclusions:**

Various serious complications can occur during locoregional anesthesia techniques in ophthalmic surgery. The mastering and perfecting of these techniques by practitioners and compliance with safety standards in anesthesia are the only way to guarantee the prevention of such complications.

## Introduction

In ophthalmic surgery several anesthetic techniques are possible [1-3]. The use of locoregional anesthesia (LRA) is more practiced at the expense of general anesthesia. These techniques have had major developments in recent years [4-6].

From these techniques, peribulbar anesthesia (PBA) has been very successful over the retrobulbar anesthesia seen its effectiveness and safety [7,8]. Despite this success, varying complications during PBA have been described [9-11]. Of these, convulsions are a serious complication [12-14].

Through a clinical case of a convulsion during PBA and a literature review, we discuss the mechanisms involved in this complication and possible means of prevention.

## Case presentation

A 70-year-old, 83kg African man was scheduled for cataract surgery and lens implant for his right eye. His medical history included hypertension, diabetes mellitus and gall bladder surgery under general anesthesia. There was no surgery on his contralateral eye. His medications included amlodipine, insulin, antiplatelets and statins. There were no personal or family antecedents of allergy, epilepsy or taking food or toxic drug.

A routine preoperative evaluation noted a blood pressure (BP) of 149/78mmHg, a heart rate (HR) of 81 beats/minute and arterial oxygen saturation (SpO_2_) of 99%.

A clinical examination did not show dyspnea or angina. No abnormalities were detected on an electrocardiogram and a chest X-ray. In laboratory tests, his blood glucose was 158mg/dL, urea plasma was 0.49g/L and creatinine was 13mg/L. In the anesthetic consultation, it was decided to continue all treatments and to stop insulin on the morning of surgery. After a preoperative fasting of 6 hours, premedication by hydroxyzine (75mg), and oral administration of prophylactic antibiotic (ciprofloxacin 1g), the patient was admitted to the operating room where monitoring including BP, HR and SpO_2_ was installed (Zeus Infinity Empowered Dräger Medical AG & Co. KG. Lübeck, Germany). A peripheral venous catheter 18g was inserted in his right hand and powered by an infusion of saline serum (0.9%).

After swabbing and explanation of the technique to the patient, we proceeded to PBA with two injections in primary gaze position. The anesthetic mixture contained lidocaine 2% and bupivacaine 0.5%. The needle used was 25GA, 19mm, ¾ inch (Alcon® Surgical Malmaison Cedex, France). The first injection was performed in his lower temporal peribulbar space with 5mL of mixture; the second injection was performed with 3mL of mixture in his upper nasal peribulbar space. These injections were performed after a negative aspiration test and followed by manual compression of his globe for 5 minutes. During injections no change in BP, HR or SpO_2_ was noted. Five minutes after PBA, his BP increased to 209/115mmHg but his HR did not change (85 beats/minute) neither did his SpO_2_ (99%). This hypertension lasted 3 minutes; three bolus of nicardipine (3.0mg) were required to reduce his BP to 134/56mmHg. One minute after, he had generalized tonic–clonic seizures. He was placed in a lateral position and 3mg of midazolam was administered. Tracheal intubation was performed successfully after a bolus of propofol (150mg) and fentanyl (150μg) without muscle relaxants. The controlled ventilation was made with sedation by isoflurane 1%. His capillary blood glucose was 170mg/dL, axillary temperature was 36.5°C, and his serum electrolytes were normal. He recovered spontaneous ventilation 1.5 hours later. A neurological examination noted no deficit. Extubation was performed 15 minutes later without incident. A brain computed tomography and electroencephalogram were unremarkable. He was discharged on the second day and operated on 1 month later under general anesthesia.

## Discussion

There has been much development in LRA in recent years from a technical standpoint and in equipment. In ophthalmic surgery, PBA has been very successful over the retrobulbar anesthesia seen its effectiveness and safety [7,8]. Despite this success, varying complications during PBA have been described. Of these, convulsions represent a serious complication [12-14].

During surgery under LRA, convulsions may have several causes such as hypoglycemia, medication errors, stroke, severe hypoxia caused by deep sedation or following a cardiac arrest complicating cardiac ocular reflex and central nervous system intoxication by spread of local anesthetic agent (LAA) (Table 1). Several mechanisms may explain the spread of LAA used in PBA to brain structures causing various neurological signs.

**Table 1:** Perioperative causes of convulsions and their management and prevention

**Causes**	**Symptoms**	**Management**	**Prevention**
Oculocardiac reflex	Bradycardia/cardiac arrest	Release of muscle tension	Gentle muscle tractions
		Injection of atropine	Injection of atropine
Drug errors	Tachycardia/bradycardia/convulsions… (depends on type of drugs injected)	Symptomatic treatment	Labeling of syringes
			Verification of syringes before injection
Hypoglycemia	Sweating, drowsiness, confusion, abnormal behavior … convulsions	Administration of serum glucose (10%)	No taking of hypoglycemic medications the morning of surgery
			Perioperative monitoring of blood glucose
Hypoxia	Desaturation, consciousness disorders … convulsions.	Oxygen therapy, liberation of superior airways, intubation	Monitoring of oxygen saturation
			Systematic supplemental oxygen during sedation
			Monitoring of sedation
Stroke	Consciousness disorders, respiratory distress, hemodynamic instability, convulsions	Control of airway, control of hemodynamics parameters	Management of cardiovascular drugs
			Neurological monitoring
			Maintaining a stable hemodynamic and normal oxygenation
Toxicity of local anesthetics	Somnolence, diplopia, shivering,	Oxygen therapy	Aspiration before injection
	Difficulty speaking, arrhythmias	Airway liberation	Reduction of local anesthetic doses
	Convulsions, coma	Anticonvulsants,	Use of ultrasound
		Fat emulsion	

The first mechanism is an inadvertent intra-arterial injection in the ophthalmic artery or its branches. The injection pressure can reverse the direction of blood flow in the artery and the anesthetic solution flows back into the internal carotid artery and is delivered to the thalamus and other midbrain structures [14-16].

Indeed the ophthalmic artery is in an abnormal position inferior to the optic nerve in 15% of cases, this exposes inadvertent intra-arterial injection during a PBA [16]. In this case, the direct exposure of brain structures to a low volume of LAA is similar to intoxication after inadvertent peripheral intra-arterial injection of a large volume of LAA, thereby clinical presentation is similar. Clinical signs have rapid onset and can range from loss of consciousness to cardiac arrest. Convulsions may be present or replaced by an electric silence [17].

The second mechanism is inadvertent brainstem anesthesia [18-21]. The optic nerve is surrounded by three envelopes, which are an extension of the cerebral meninges. Studies have shown that there is communication between the central cerebral structures and the optic nerve [22,23]. Thereby an accidental injection through these envelopes causing a diffusion of LAA under arachnoid or subdural space to brain structures can cause a direct intoxication of brain structures. The main risk factors for this iatrogenic injection are the size of the needle used and the position of the eye at the time of realization of the PBA.

During PBA, with the globe in superonasal gaze, the optic nerve is in close proximity to the introduced needle [10]. In this superonasal gaze position, the risk of puncture of the envelope of the optic nerve is important with a diffusion of LAA into the brain structures. However, in a primary gaze position, the risk of accidental puncture of the optic nerve is low.

Although the distance between the temporal region and lower optical foramen is between 42mm and 54mm, the use of shorter needles between 16mm and 25mm and a higher volume of LAA allows, after a period of time, complete anesthesia. The onset of action is explained by the diffusion of LAA at the site of action. With these needles the risk of puncture of the optic nerve is low in comparison with the use of long needles of 31mm or more, which provide an immediate anesthesia with a low volume of LAA, but increase the risk of puncture of the optic nerve [24,25]. Another mechanism that can be evoked is the absorption of LAA by the arachnoid villi and spread to cerebral structures [26]. This absorption is favored by manual compression and the use of hyaluronidase.

In our patient, there was no hypoglycemia (capillary blood glucose was 170mg/dL), no medication error in the verification of injected drugs, and no metabolic disorders in laboratory analysis. There was no neurological deficit after awakening and his postoperative brain computed tomography was normal eliminating a cerebral stroke. Thus, the convulsions were very probably related to PBA technique.

The delayed onset of convulsions after PBA and recovery of spontaneous ventilation after 1 hour are in favor of the second mechanism (inadvertent brainstem anesthesia). Using a 19mm needle in our case, we cannot exclude the possibility of this mechanism. Two elements are against the first mechanism of intra-arterial injection: negativity of needle aspiration test and delayed onset of symptoms. Whatever the mechanism involved in this complication, prevention is necessary to avoid the occurrence of such complications.

During these last years, surgeons increasingly perform LRA in ophthalmic surgery [27]; this raises the question of the necessity of the presence of anesthetists in the operative room. This presence of anesthetists always remains required during the LRA for the management of complications.

The respect of standards in anesthetic safety in ophthalmic surgery is mandatory [28,29]. The type of monitoring used during ophthalmic local anesthesia should be similar to that used during general anesthesia and all technical and human resources must be available, in the operative room, to deal with the occurrence of any complications [30]. The other elements of prevention are: the systematic practice of needle test aspiration before each injection, maintaining the position of the globe in a neutral position at the completion of the PBA, and the use of needles no longer than 25mm.

The use of ultrasound in LRA in ophthalmic surgery could help guide the direction of the needle, see the injection sites, reduce the volume of injected LAA and reduce the complication rate and improve the safety and performance of these techniques. These benefits have already been demonstrated in LRA for other surgeries; in ophthalmic surgery, the use of ultrasound for the realization of LRA is in full development [31-33].

## Conclusions

LRA techniques in ophthalmic surgery should not be trivialized. Various serious complications can occur during LRA techniques in ophthalmic surgery.

The mastering and perfecting of these techniques by practitioners and compliance with safety standards in anesthesia are the only way to guarantee the prevention of such complications.

## Consent

Written informed consent was obtained from the patient for publication of this case report and accompanying images. A copy of the written consent is available for review by the Editor-in-Chief of this journal.

## Abbreviations

BP: Blood pressure; HR: Heart rate; LAA: Local anesthetic agent; LRA: Locoregional anesthesia; PBA: Peribulbar anesthesia; SpO_2_: Arterial oxygen saturation.

## Competing interests

The authors declare that they have no competing interests.

## Authors’ contributions

MB, NB and AH analyzed and interpreted the patient data. MB and NB were major contributors in writing the manuscript. HB, CH and HA made the final corrections. All authors read and approved the final manuscript.
